# Integrated Analysis of Key Differentially Expressed Genes Identifies DBN1 as a Predictive Marker of Response to Endocrine Therapy in Luminal Breast Cancer

**DOI:** 10.3390/cancers12061549

**Published:** 2020-06-12

**Authors:** Lutfi H. Alfarsi, Rokaya El Ansari, Brendah K. Masisi, Ruth Parks, Omar J Mohammed, Ian O. Ellis, Emad A. Rakha, Andrew R. Green

**Affiliations:** 1 Nottingham Breast Cancer Research Centre, Division of Cancer and Stem Cells, School of Medicine, University of Nottingham Biodiscovery Institute, University Park, Nottingham NG7 2RD, UK; lutfi.alfarsi@nottingham.ac.uk (L.H.A.); msxre2@exmail.nottingham.ac.uk (R.E.A.); msxbkma@exmail.nottingham.ac.uk (B.K.M.); msarp7@exmail.nottingham.ac.uk (R.P.); msaom1@exmail.nottingham.ac.uk (O.J.M.); mrzie@exmail.nottingham.ac.uk (I.O.E.); mrzear1@exmail.nottingham.ac.uk (E.A.R.); 2 Cellular Pathology, Nottingham University Hospitals NHS Trust, Nottingham City Hospital, Hucknall Road, Nottingham NG5 1PB, UK

**Keywords:** DBN1, oestrogen receptor, breast cancer, endocrine resistance, predictive biomarker

## Abstract

Endocrine therapy is the mainstay of adjuvant treatment for patients with luminal breast cancer. Despite ongoing advances in endocrine therapy to date, a proportion of patients ultimately develop endocrine resistance, resulting in failure of therapy and poor prognosis. Therefore, as part of the growing concept of personalised medicine, the need for identification of predictive markers of endocrine therapy response at an early stage, is recognised. The METABRIC series was used to identify differentially expressed genes (DEGs) in term of response to adjuvant endocrine therapy. Drebrin 1 (DBN1) was identified as a key DEG associated with response to hormone treatment. Next, large, well-characterised cohorts of primary luminal breast cancer with long-term follow-up were assessed at the mRNA and protein levels for the value of DBN1 as a prognostic marker in luminal breast cancer, as well as its potential for predicting the benefit of endocrine therapy. DBN1 positivity was associated with aggressive clinicopathological variables and poor patient outcomes. Importantly, high DBN1 expression predicted relapse patients who were subject to adjuvant endocrine treatment. Our results further demonstrate that DBN1 is an independent prognostic marker in luminal breast cancer. Its association with the response to endocrine therapy and outcome provides evidence for DBN1 as a potential biomarker in luminal breast cancer, particularly for the benefit of endocrine treatment. Further functional investigations into the mechanisms underlying sensitivity to endocrine therapy is required.

## 1. Introduction

Breast cancer exhibits significant heterogeneity with different molecular subtypes, and the most common subtype of the disease is luminal, Oestrogen Receptor-positive (ER+) tumours. Endocrine therapy has been widely used in clinical practice as adjuvant treatment for this subtype. Although the outcome of patients with luminal tumours have markedly improved with endocrine therapy [1], around 30–50% of patients with luminal breast cancer relapse despite treatment [2]. Therefore, research priorities are required to better identify biomarkers to aid clinician decision-making.

Advancement in microarray and high-throughput sequencing technologies have provided an efficient tool for deciphering key molecular mechanisms in tumourigenesis. Integrated bioinformatics approaches and gene expression data are useful in screening for differentially expressed genes (DEGs), which may aid in elucidating the molecular mechanisms underlying endocrine therapy resistance in luminal breast cancer, and to identify the novel and effective molecular markers. Therefore, in this study we sought to screen for the DEGs through analysis of the transcriptomic profiles of the Molecular Taxonomy of Breast Cancer International Consortium (METABRIC) cohort to identify potential predictive markers associated with endocrine therapy response and, subsequently, validate the protein expression of a putative marker in a large annotated cohort of luminal breast cancer.

## 2. Results

### 2.1. Identification of DEGs

**Figure 1:**
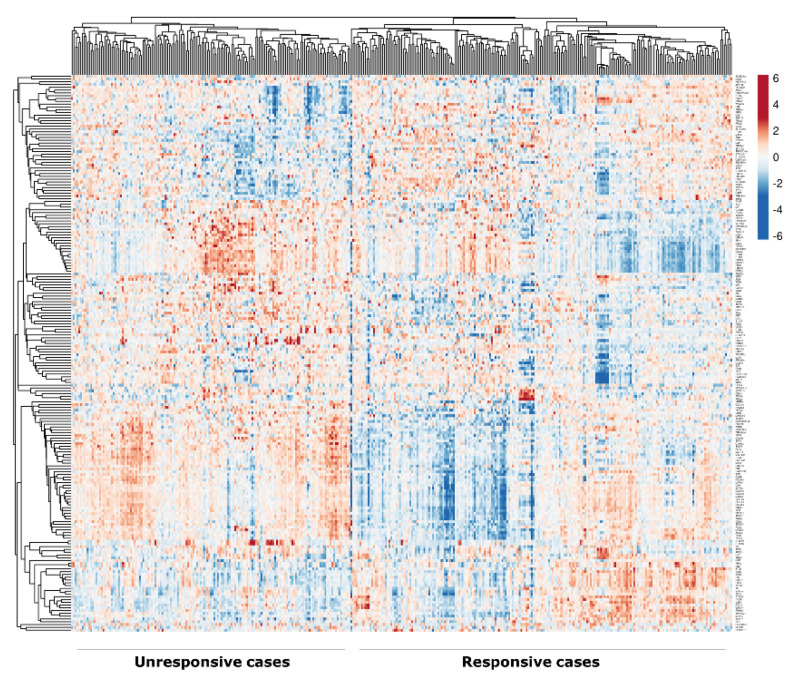
Heatmap of the differentially expressed genes (DEGs) associated with response to endocrine treatment, generated using the ClustVis web tool (Tartu, Estonia). Rows and columns were clustered using correlation distances and average linkage. Red and blue colours indicate high and low, respectively.

Based on the rank of WAD analysis for the top DEGs, 200 genes were identified between the unresponsive (defined as patients who received endocrine therapy as the only adjuvant treatment but had recurrence and/or distant metastasis over the course of follow-up (mean = 75 months)) and responsive (defined as patients who received adjuvant endocrine treatment as the only adjuvant treatment and had no recurrence, distant metastasis or death from breast cancer over the course of follow-up (mean = 117 months)) cases of luminal breast cancer treated with adjuvant endocrine therapy, of which 129 were upregulated in the unresponsive cases and 71 were downregulated (Table S1), with a heatmap representing the pattern of gene expression between the responsive and unresponsive cases to endocrine therapy (Figure 1). Analysis of the DEGs list identified the enriched pathways associated with unresponsive cases to endocrine therapy, including the integrin signalling pathway (*p* = 0.01) and Platelet-derived Growth Factor (PDGF) pathway (*p* = 0.03), Table S2.

### 2.2. DBN1 Expression in Luminal Breast Cancer

Derbrin 1 (DBN1) was identified as one of the highly ranked genes and was the most highly associated with relapse in patients who were treated with adjuvant endocrine therapy using Kaplan–Meier survival plots.

**Figure 2:**
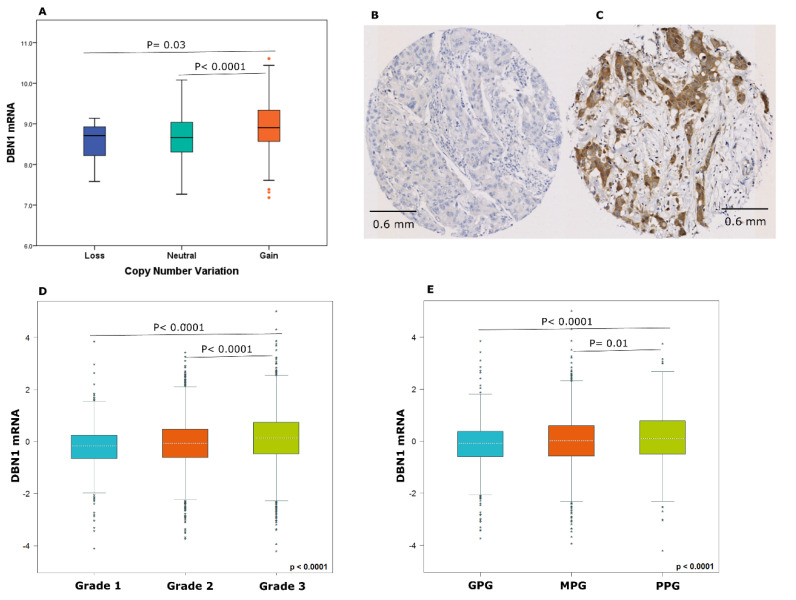
(**A**) DBN1 copy number variation and relationship with mRNA expression in the METABRIC cohort using one-way analysis of variance and the post-hoc Tukey test. Representative immunostaining images of invasive breast cancer using IHC (**B**) negative and (**C**) positive for DBN1 protein expression. DBN1 mRNA expression and its association with the clinicopathological parameters. (**D**) tumour grade and (**E**) the Nottingham Prognostic Index, using the bc-GenExMiner dataset.

A total of 70% of the METABRIC cohort were aged > 50 years and 72% were treated with endocrine therapy. DBN1 mRNA was highly expressed in 37% of luminal breast cancers from the METABRIC cohort, and its copy number gain was observed in 7.7% of cases, whereas 1.6% showed a copy number loss. Significant association was observed between DBN1 copy number variation and DBN1 mRNA expression (*p* < 0.05; Figure 2A).

For the Nottingham cohort, 71% were aged > 50 years and 42% were treated with endocrine therapy. DBN1 protein expression was predominantly expressed in the cytoplasm of invasive breast cancer cells, with intensity levels varying from absent to high (Figure 2B,C). After excluding the uninformative TMA cores from the study, 584 tumours were available for the assessment. DBN1 expression was dichotomised into low and high groups based on an H-score ≥ 95, and its high expression was observed in 45% of the cases.

Next, the association of DBN1 mRNA expression with the clinicopathological parameters in luminal breast cancer was investigated using the METABRIC cohort and validated by using the bc-GenExMiner dataset. There was a positive association between DBN1 mRNA expression and tumour grade as well as high risk patients (Nottingham Prognostic Index (NPI ≥ 3.4) using the METABRIC cohort, Table S3, and bc-GenExMiner dataset (*p* < 0.05; Figure 2D,E). The association between DBN1 protein expression and other clinicopathological parameters did not reach statistical significance, Table S3.

### 2.3. Clinical Significance of DBN1

**Figure 3:**
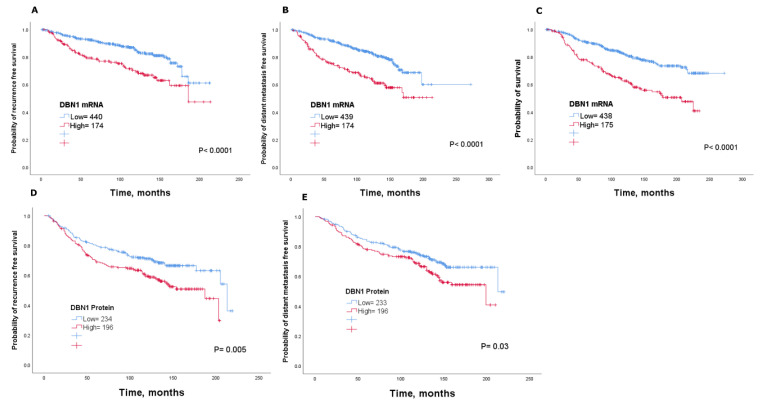
Association of DBN1 mRNA and DBN1 protein with patient outcome in all luminal breast cancer irrespective of treatment using the METABRIC cohort: (**A**) recurrence, (**B**) distant metastasis and (**C**) survival; and the Nottingham cohort: (**D**) recurrence and (**E**) distant metastasis.

In terms of clinical outcome, patients with higher *DBN1* tumour levels in the METABRIC cohort had an unfavourable outcome compared with those patients with low *DBN1* expression (*p* < 0.05; Figure 3A–C). Consistent with this finding, high DBN1 mRNA expression was significantly associated with high risk of recurrence, distant metastasis and death from breast cancer using KM-Plotter (*p* < 0.05; Figure S1A–C) and bc-GenExMiner v4.3 (*p* < 0.05; Figure S1D,E). Multivariate analysis using the METABRIC cohort showed that DBN1 mRNA expression is independent of tumour size, nodal stage and tumour grade in predicting recurrence. Indeed, analysis showed that DBN1 mRNA, along with tumour size and grade, independently predicted the distant metastasis and survival (*p* < 0.05; Table 1).

To directly interrogate this association at the protein level, we performed a Kaplan–Meier survival analysis for the DBN1 protein. Results revealed that patients with high DBN1 protein expression had adverse outcomes compared to patients with low DBN1 expression (*p* < 0.05; Figure 3D,E). Using multivariate Cox regression analysis, including tumour size, nodal stage and tumour grade, DBN1 protein expression, along with tumour size and nodal stage, was an independent prognostic marker for recurrence (*p* < 0.05; Table 1). Within the different molecular subtypes of luminal tumours, high DBN1 protein expression was associated with shorter survival in ER+ high proliferation/luminal B tumours (*p* < 0.05; Figure S2), but not for ER+ low proliferation/luminal A subtype (*p* =  0.7; Figure S3). There was no association between DBN1 expression and survival in triple-negative breast cancers (*p* > 0.05; Figure S4).

### 2.4. DBN1 Expression Predicts Poor Response in Endocrine-Treated Patients

**Figure 4:**
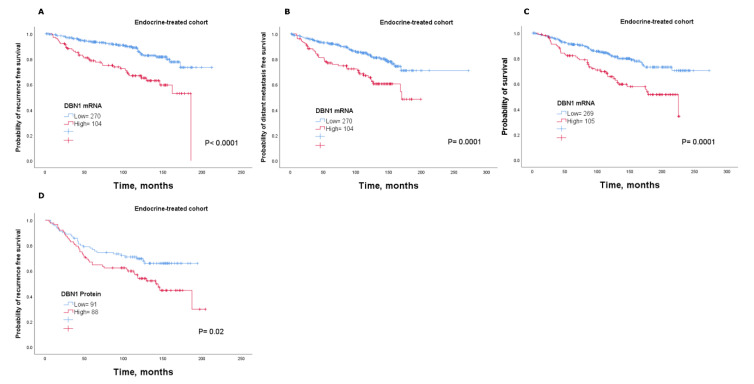
Association of DBN1 mRNA and DBN1 protein with patient outcome in luminal breast cancer who received adjuvant endocrine treatment only using the METABRIC cohort: (**A**) recurrence, (**B**) distant metastasis and (**C**) survival; and using the Nottingham cohort: (**D**) recurrence.

The ability of DBN1 in predicting the response of endocrine therapy was tested by analysing its association with clinical outcome in a subgroup of patients with luminal tumours who received adjuvant endocrine therapy alone. The analysis revealed that patients with tumours that highly expressed DBN1 mRNA were associated with a shorter time to recurrence, distant metastasis and survival than those with low *DBN1* expression (*p* < 0.05; Figure 4A–C). This was validated using the KM-Plotter dataset where high DBN1 mRNA expression was significantly associated with poor outcome after endocrine treatment (*p* < 0.05; Figure S5).

DBN1 protein results were consistent with the mRNA, whereby patients who were subject to endocrine treatment and with high expression had a higher risk of recurrence compared to patients with low expression (*p* < 0.05; Figure 4D). Cox regression analysis revealed that both the DBN1 mRNA and DBN1 protein levels are predictive markers for a higher risk of relapse in the subgroup of patients who were treated with endocrine therapy alone (*p* < 0.05; Table 2).

## 3. Discussion

Luminal breast cancer is not only heterogeneous in clinical presentations but also varied in terms of prognostic implications and treatment response. Although adjuvant endocrine therapy offers prospects for improved survival, the lack of benefit of this treatment in some patients remains a major issue, which has continued to attract attention both experimentally and clinically. Therefore, novel and reliable biomarkers of the response to endocrine treatment are of urgent need. In this study, we used gene expression profiling of the METABRIC cohort and various bioinformatics analysis to identify the DEGs related to poor outcome in luminal breast cancer and potentially benefitting from endocrine therapy.

In this study, a number of DEGs were identified, which have prognostic roles in luminal breast cancer, particularly in predicting the response to endocrine therapy in breast cancer. Better understanding of the pathways involved in endocrine therapy resistance may provide important clues to the mechanisms of resistance that might involve activation of escape pathways that can enhanced the proliferation and growth of the luminal tumours. In light of this, our results of the Panther pathway analysis demonstrate that the integrin signalling pathway was the most enriched pathway associated with the DEGs within the cases unresponsive to endocrine therapy. In support of our finding, previous studies have linked the integrin signalling pathway in breast tumours to cell migration and invasion, and contribution to endocrine resistance via interaction with the tumour microenvironment in an integrin-dependent manner [3,4,5]. Of interest, *MYC* and *CCND1* were found among the identified DEGs that were upregulated in the unresponsive cases to endocrine therapy. This is in line with previous studies that showed overexpression of the cell cycle regulators *MYC* and *CCND1* are associated with endocrine resistance [6,7], where it is well-recognised that upregulation of the cell cycle regulators, especially those controlling G1 phase progression, can causes endocrine resistance [8]. Additionally, the amino acid transporter *SLC7A5* was also among the upregulated DEGs in the unresponsive cases identified in this study. In support of this finding, we have previously along with others reported that SLC7A5 high expression correlates with poor clinical outcome and poor response to endocrine therapy in patients with luminal breast cancer [9,10,11]. Altogether, the above findings indicate a good reliability of the analysis performed in identifying the DEGs that are associated with prognosis in luminal breast cancer and sensitivity to endocrine treatment.

As DBN1 was a key DEG in predicting response to adjuvant endocrine therapy, the clinical impact of its expression on luminal breast cancers was investigated. Specifically, its role as a predictive marker to determine the sensitivity to endocrine therapy was addressed. DBN1 is an actin-binding protein that was initially associated with the process of neuronal growth [12,13]. However, DBN1 expression has been recently found in a wide variety of non-neuronal cells, including cancer cells [14,15,16,17,18]. Recently, it has been shown that inhibition of DBN1, with a small molecule inhibitor of drebrin binding to actin filaments, reduced the invasion of prostate cancer cell lines in 3D in vitro assays [19]. Further, DBN1 was demonstrated to bind and regulate the chemokine receptor CXCR4 recruitment to the immune synapse [20]. Interestingly, several studies have shown that CXCR4 has a key role in stimulating the chemotactic and invasive behaviour of breast cancer cells, and blocking CXCR4 prevents breast cancer metastatic spread in vitro and in vivo [21,22,23]. These findings, therefore, suggest a key role for DBN1 in breast cancer cell invasion. However, to date, the clinical significance of DBN1 in luminal breast cancer remains unclear. In this study, we found that elevated DBN1 expression was associated with aggressive clinicopathological variables. These findings suggesting a potential role for DBN1 in tumorigenesis of luminal breast cancer.

In a recent study of non-small cell lung cancer, DBN1 was reported as a genomic marker, exhibiting potential clinical utility for risk stratification of Stage I–III [14]. Indeed, another study showed that cases with high DBN1 expression had a significantly poorer prognosis than those with low expression, suggesting it could act as a prognostic biomarker in lung adenocarcinoma [24]. In addition, our results indicated that expression of DBN1 has important effects on the clinical outcome of patients with luminal breast tumours. In the mRNA cohorts, high *DBN1* expression was associated with poor breast cancer specific survival and recurrence. At the protein levels, high cytoplasmic DBN1 expression was associated with high risk of recurrence. Regarding luminal breast cancer subtypes, DBN1 protein expression was associated with poor patient outcome in the ER+ high proliferation/luminal B subtype. The triple-negative breast cancer subtype showed no significant association between DBN1 expression and patient outcome. To the best of our knowledge, this the first study to investigate the prognostic utility of DBN1 in luminal breast cancer using large clinical data sets with long-term follow up, which suggest DBN1 as a possible prognostic marker in patients with luminal tumours.

Cell motility and invasion are important aspects of cancer metastasis that involve cell morphology changes that are associated with dynamic remodelling of the actin cytoskeleton. Interestingly, DBN1 is thought to be a regulator of actin filament assembly, thereby contributing to cell motility and morphology [13], suggesting that DBN1 could deliver this role during cancer metastasis. Additionally, it has been shown that knockdown of DBN1 decreased the invasion and migration of glioma cells, while DBN1 overexpression leads to alterations in cell morphology and induces increased invasiveness in vitro [15]. In prostate cancer, it has been shown that knockdown of DBN1 decreases the invasion of cell lines in 3D in vitro assays, whereas DBN1 overexpression enhanced it [19]. In the present study, we found that patients with high DBN1 expression was associated with a higher risk of distant metastasis than those with low expression, which supports the idea that DBN1 might associate with the invasive and migratory behaviour of tumours.

Moreover, this study has revealed that DBN1 expression had a significant association with sensitivity to endocrine therapy. High DBN1 mRNA expression was significantly associated with increased risk of death, recurrence and distant metastasis among the endocrine-treated patients with luminal breast cancer. Indeed, our results indicate that patients with higher cytoplasmic DBN1 expression are less likely to benefit from endocrine therapy. However, the exact molecular mechanisms of the effects of DBN1 on endocrine sensitivity remain unclear and therefore need further investigation. Currently, there remains limited biomarkers that have great predictive value above the ER and progesterone receptor to facilitate optimal endocrine treatment selection in the adjuvant setting. Despite the use of multigene signatures assays in some cases, these tools only help in risk stratification and the likelihood of benefit from chemotherapy, which are unable to predict the response to endocrine therapy in the early stage of luminal breast cancer [25,26]. Our findings suggest that DBN1 expression could be used as a prognostic marker in luminal breast cancer and to better identify the appropriate patients whose tumours are most likely to benefit from endocrine therapy. These data require further confirmation in sensitive and tamoxifen-resistant model systems prior to multicentre studies of pre-clinical and clinical trials, to validate the clinical value of DBN1 as a predictive biomarker. Particularly as the exact molecular mechanisms of the effects of DBN1 on endocrine sensitivity remains unclear.

## 4. Materials and Methods

### 4.1. Ethical Approval

This study was performed according to the REMARK guidelines for tumour prognostic studies [27] and approved by the Nottingham Research Ethics Committee 2 under the title “Development of a molecular genetic classification of breast cancer” (REC202313 April 2019).

### 4.2. DEGs Analysis

The weighted average difference (WAD) method [28] in R language was used to identify the DEGs between the unresponsive (patients who received endocrine therapy (Tamoxifen) as the only adjuvant treatment but had recurrence and/or distant metastasis, *n* = 89) and responsive cases (patients who received adjuvant endocrine treatment and had no recurrence, distant metastasis or death from breast cancer, *n* = 292) using the METABRIC cohort. This cohort contains 1506 samples of primary luminal (ER+/HER2-negative) invasive breast carcinomas before adjuvant treatment. The experimental assays and analytical methods used is as previously described [29]. The characteristics of this cohort are summarised in Table S4. Selection of DEGs was based on the WAD ranking. The heatmap of the identified DEGs associated with the response to endocrine treatment was generated using the ClustVis web tool [30]. To determine the most enriched categories of pathways for the top DEGs, the list of genes was uploaded to the WEB-based Gene SeT AnaLysis Toolkit (WebGestalt) website [31]. The analysis was performed by choosing a “*Homo sapiens*” in the menu, Gene Set Enrichment Analysis in the method and selecting the functional database category as the pathway with the option of Panther pathway analysis; a *p* value of < 0.05 was considered statistically significant. The study design and selection criteria of DBN1 are illustrated in (Figure S6).

### 4.3. mRNA Expression Cohorts

The METABRIC cohort (*n* = 1980) was used to identify the key DEGs that are associated with a response to endocrine therapy in luminal breast cancer. Additionally, this cohort was used to investigate the prognostic value of *DBN1* in predicting the clinical outcome for patients with luminal tumours and thus the benefit of endocrine treatment.

The Kaplan–Meier Plotter Breast Cancer (KM-Plotter) dataset (*n* = 3951) [32] was used as a validation cohort for the prognostic and predictive value of *DBN1*. Breast Cancer Gene-Expression Miner v4.3 (bc-GenExMiner v4.3) (*n* = 4842) [33] was used to analyse the prognostic value and test its association with the clinicopathological parameters in this dataset.

### 4.4. Protein Expression Analysis

DBN1 protein expression was assessed in a well-characterised series of luminal primary invasive breast cancer patients (*n* = 436), with long-term follow-up. Patients presented at the Nottingham City Hospital (1989–2006), as previously described [34]. The characteristics of this cohort are summarised in Table S2.

The specificity of DBN1 antibody (Ab60933, Abcam, UK) was validated prior to the staining by Western blotting using MCF7 human breast cancer cell lysate (American Type Culture Collection; Rockville, MD, USA), as previously described [34]. The specificity of the DBN1 antibody was observed with a single band at the predicted size of approximately 100 kDa (Figure S7). Immunohistochemistry (IHC) was used to assess DBN1 protein expression on 4-μm tissue microarray sections using Novolink polymer detection system (RE7150-K, Leica Biosystems, UK), as previously described [34] using the DBN1 antibody at a 1:1000 dilution. Evaluation of cytoplasmic staining for DBN1 in invasive tumours cells was based on a semi-quantitative assessment of invasive tumour cells using a modified histochemical score (H-score) [35]. Tissue microarray cores were only assessed if the invasive tumour burden was > 15%.

### 4.5. Clinical Outcomes Data

Clinical outcomes included breast cancer specific survival defined as the time in months from the diagnosis to the date of death from breast cancer (METABRIC cohort: mean = 121 months, range 1–272; Nottingham cohort: mean = 164 months, range 1–307). Recurrence-free survival was defined as the time in months from diagnosis until developing local or regional recurrence (mean = 101 months, range 1–213; Nottingham cohort: mean = 110 months, range 2–247). Distant metastasis-free survival was defined as the time in months from diagnosis until developing distant metastasis (mean = 104 months, range 1–272; Nottingham cohort: mean = 121 months, range 2–247).

### 4.6. Statistical Analysis

SPSS statistical software (version 25, Chicago, IL, USA) was used for data analysis. The Chi-square test was performed to evaluate the association between DBN1 expression and the clinicopathological parameters. A *t*-test was used to assess the mean difference between two groups. One-way analysis of variance (ANOVA) with the post-hoc Tukey multiple comparison test was used to assess differences in means between three or more groups. Kaplan–Meier survival curves were used to assess the association of DBN1 expression with clinical outcome. Cox regression analysis was used to evaluate the independent prognostic significance of DBN1 expression. The Benjamini–Hochberg procedure for multiple test correction was performed. The dichotomisation of DBN1 mRNA and protein expression into low and high groups was determined using X-Tile (X-Tile Bioinformatics Software, Yale University, version 3.6.1, NH, USA) based on the prediction of recurrence-free survival. A *p* value of < 0.05 was considered significant.

## 5. Conclusions

The current study provides definitive evidence that DBN1 is an independent prognostic marker of poor clinical outcome in patients with luminal breast cancer. Most importantly, our study has clearly showed that DBN1 expression is a potential predictive marker of a lack of benefit from endocrine therapy and its measurement could aid clinician decision-making.

## Figures and Tables

**Table 1:** Multivariate cox analysis of the associations between DBN1 expression and the clinicopathological parameters in all luminal breast cancer irrespective of treatment.

**METABRIC Cohort**				
**Parameters**	**Recurrence-Free Survival**			
	HR (95% CI)	*p*		*p* *
*DBN1* Tumour size Tumour grade Nodal stage	2.2 (1.4–3.5) 1.3 (0.8–2.1) 1.2 (0.9–1.8) 1.1 (0.7–1.5)	0.0003 0.17 0.14 0.5		0.001 0.2 0.3 0.6
**Parameters**	**Distant Metastasis-Free Survival**			
	HR (95% CI)	*p*		*p* *
*DBN1* Tumour size Tumour grade Nodal stage	2.9 (1.8–4.8) 2.0 (1.1–3.3) 1.3 (0.9–2.0) 1.3 (0.9–2.0)	0.00001 0.008 0.1 0.09		0.0001 0.02 0.12 0.1
**Parameters**	**Breast Cancer Specific Survival**			
	HR (95% CI)	*p*		*p* *
*DBN1* Tumour size Tumour grade Nodal stage	3.3 (1.9–5.8) 2.0 (1.1–3.5) 1.8 (1.1–2.9) 1.4 (0.9–2.1)	0.00001 0.01 0.008 0.09		0.0001 0.016 0.02 0.1
**Nottingham Cohort**				
**Parameters**	**Recurrence-Free Survival**			
	HR (95% CI)		*p*	*p* *
*DBN1* Tumour size Tumour grade Nodal stage	1.5 (1.1–2.0) 1.4 (1.0–2.0) 1.1 (0.9–1.5) 1.6 (1.2–2.0)		0.009 0.02 0.1 0.00005	0.02 0.03 0.12 0.0003
**Parameters**	**Distant Metastasis-Free Survival**			
	HR (95% CI)		*p*	*p* *
*DBN1* Tumour size Tumour grade Nodal stage	1.4 (1.0–1.9) 1.8 (1.3–2.7) 1.3 (1.0–1.7) 1.6 (1.3–2.1)		0.04 0.0004 0.01 0.00003	0.05 0.001 0.016 0.0002

*p* *: Adjusted *p* value.

**Table 2:** Multivariate cox analysis of the associations between DBN1 expression and clinicopathological parameters in patients with luminal breast cancer who received adjuvant endocrine treatment only.

**METABRIC Cohort**				
**Parameters**	**Recurrence-Free Survival**			
	HR (95% CI)	*p*		p *
*DBN1* Tumour size Tumour grade Nodal stage	2.5 (1.4–4.3) 1.1 (0.6–1.9) 1.1 (0.7–1.7) 1.0 (0.6–1.6)	0.001 0.6 0.5 0.8		0.005 1.0 1.2 1.0
**Parameters**	**Distant Metastasis-Free Survival**			
	HR (95% CI)	*p*		p *
*DBN1* Tumour size Tumour grade Nodal stage	2.5 (1.4–4.6) 1.4 (0.8–2.7) 1.3 (0.7–2.8) 1.4 (0.8–2.2)	0.001 0.1 0.2 0.1		0.005 0.25 0.25 0.16
**Parameters**	**Breast Cancer Specific Survival**			
	HR (95% CI)	*p*		p *
*DBN1* Tumour size Tumour grade Nodal stage	3.2 (1.6–6.1) 1.8 (0.9–3.7) 1.6 (0.9–3.0) 1.2 (0.7–2.1)	0.001 0.08 0.09 0.3		0.005 0.2 0.15 0.37
**Nottingham Cohort**				
**Parameters**	**Recurrence-Free Survival**			
	HR (95% CI)		*p*	p *
*DBN1* Tumour size Tumour grade Nodal stage	1.9 (1.1–3.1) 1.6 (1.0–2.6) 1.3 (0.9–2.0) 1.7 (1.2–2.4)		0.007 0.05 0.1 0.002	0.01 0.08 0.12 0.01

p *: Adjusted *p* value.

## Supplementary Materials

The following are available online at https://www.mdpi.com/2072-6694/12/6/1549/s1, Figure S1: Kaplan–Meier of *DBN1 mRNA* and patient outcome in luminal breast cancer, Figure S2: (**A**) Kaplan–Meier of DBN1 protein expression and survival ER+ high proliferation/Luminal B. Kaplan–Meier of *DBN1 mRNA* expression and survival in luminal B using the following datasets (**B**) METABRIC (**C**) KM-Plotter (**D**) bc-GenExMiner. (**E**) Kaplan–Meier of DBN1 protein expression and survival ER+ low proliferation/Luminal A. Kaplan–Meier of *DBN1 mRNA* expression and survival in luminal A using the following datasets (**F**) METABRIC (**G**) KM-Plotter (**H**) bc-GenExMiner, Figure S3: Kaplan–Meier of *DBN1 mRNA* expression and survival in triple negative breast cancer using the following datasets (**A**) METABRIC (**B**) KM-Plotter (**C**) bc-GenExMiner. (**D**) Kaplan–Meier of DBN1 protein expression and survival in triple negative breast cancer, Figure S4: Kaplan-Meier of *DBN1 mRNA* expression in patients with luminal breast cancer who received adjuvant endocrine treatment using KM-Plotter dataset, Figure S5: (**A**) Flow diagram illustrating the study design. (**B**) Flow diagram illustrating the filtering stages used to identify DEGs, Figure S6: Western blotting result for DBN1 expression in MCF7 breast cancer cell lysate, Figure S7: Western blotting result for DBN1 expression in MCF7 breast cancer cell lysate, Table S1: Weighted Average Difference (WAD) statistics and ranking of the top DEGs associated with unresponsive cases to endocrine treatment, Table S2: List of pathways associated with the DEGs associated with unresponsive cases to endocrine therapy, Table S3: Association of DBN1 expression and clinicopathological parameters in luminal breast cancer, Table S4: Clinicopathological characteristics of luminal breast cancer cohorts.
