# Supplementary material for: Integrated Analysis of Key Differentially Expressed Genes Identifies DBN1 as a Predictive Marker of Response to Endocrine Therapy in Luminal Breast Cancer
Source: Cancers (Basel). 2020 Jun 12;12(6):1549. doi: 10.3390/cancers12061549 (PMC7352383; doi:10.3390/cancers12061549)
Supplement: Supplementary file 1 [file cancers-12-01549-s001.pdf]

Supplementary Materials

Integrated Analysis of Key Differentially Expressed Genes Identifies DBN1 as a Predictive Marker of Response to Endocrine Therapy in Luminal Breast Cancer

Lutfi H. Alfarsi, Rokaya El Ansari, Brendah K. Masisi, Ruth Parks, Omar J Mohammed, Ian O. Ellis, Emad A. Rakha and Andrew R. Green

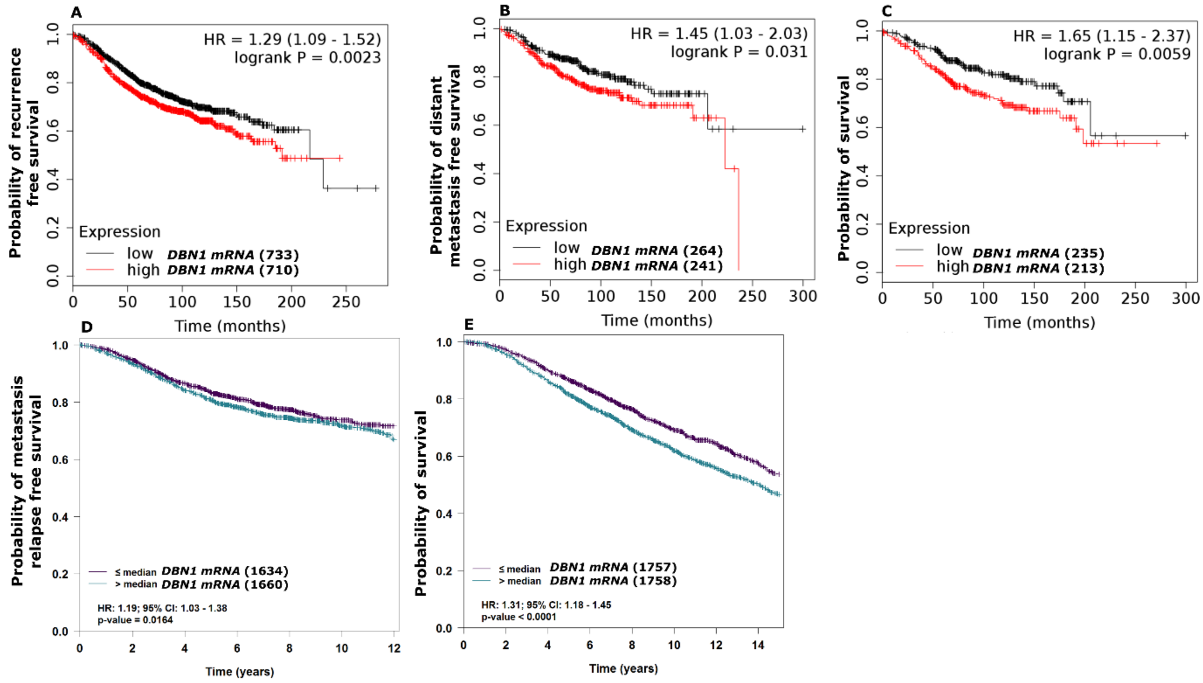

Figure S1. Kaplan–Meier of *DBN1 mRNA* and patient outcome in luminal breast cancer for (**A**) Recurrence (**C**) Distant metastasis and (**D**) Survival using KM-Plotter dataset, and for (**B**) Metastatic relapse and (**E**) Survival using bc-GenExMiner dataset.

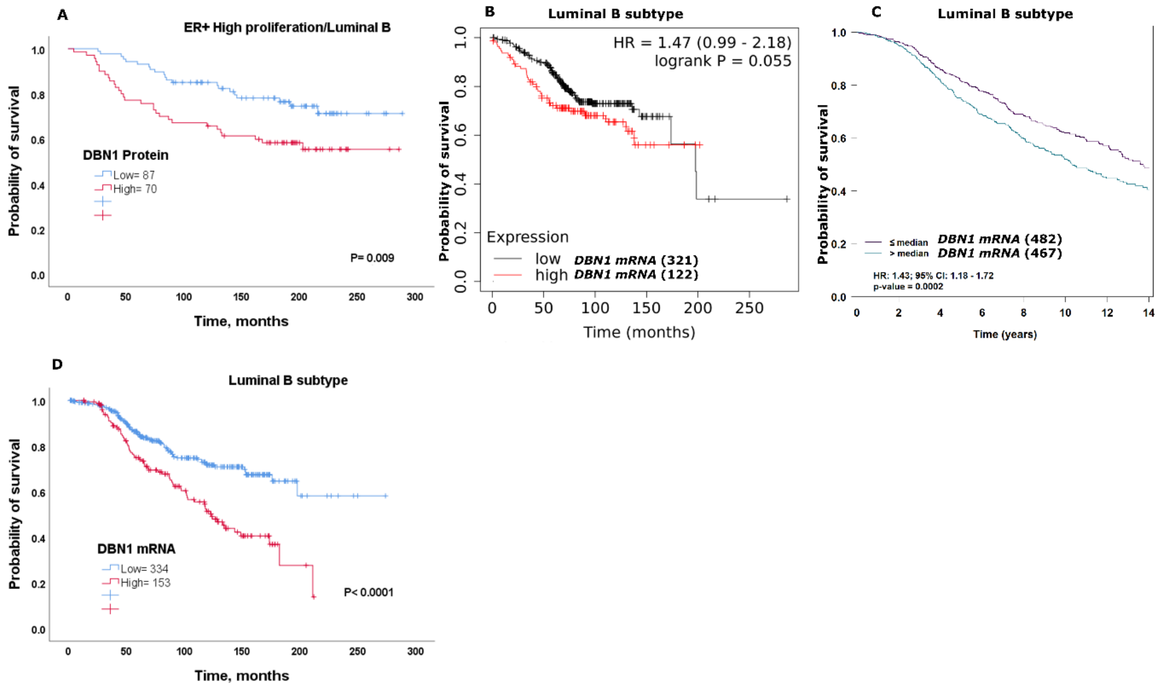

Figure S2. (**A**) Kaplan–Meier of DBN1 protein expression and survival ER+ high proliferation/Luminal B. Kaplan–Meier of *DBN1 mRNA* expression and survival in luminal B using the following datasets (**B**) METABRIC (**C**) KM-Plotter (**D**) bc-GenExMiner.

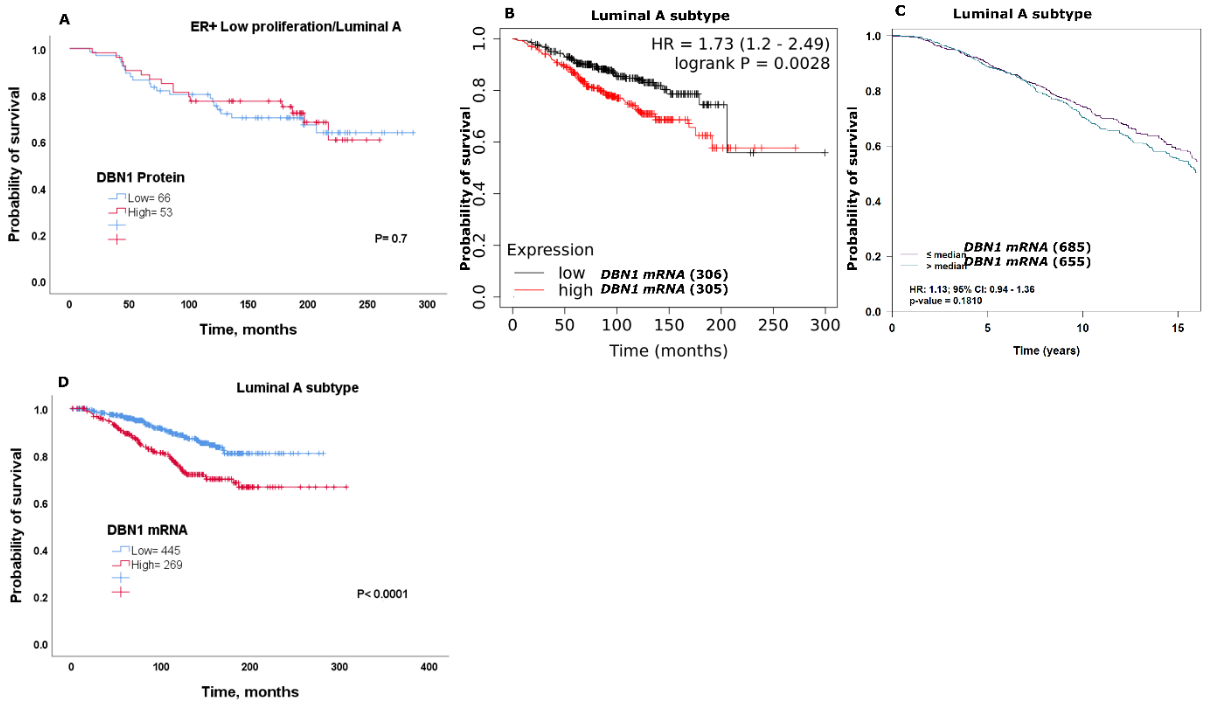

Figure S3. (**A**) Kaplan–Meier of DBN1 protein expression and survival ER+ low proliferation/Luminal A. Kaplan–Meier of *DBN1 mRNA* expression and survival in luminal A using the following datasets (**B**) METABRIC (**C**) KM-Plotter (**D**) bc-GenExMiner.

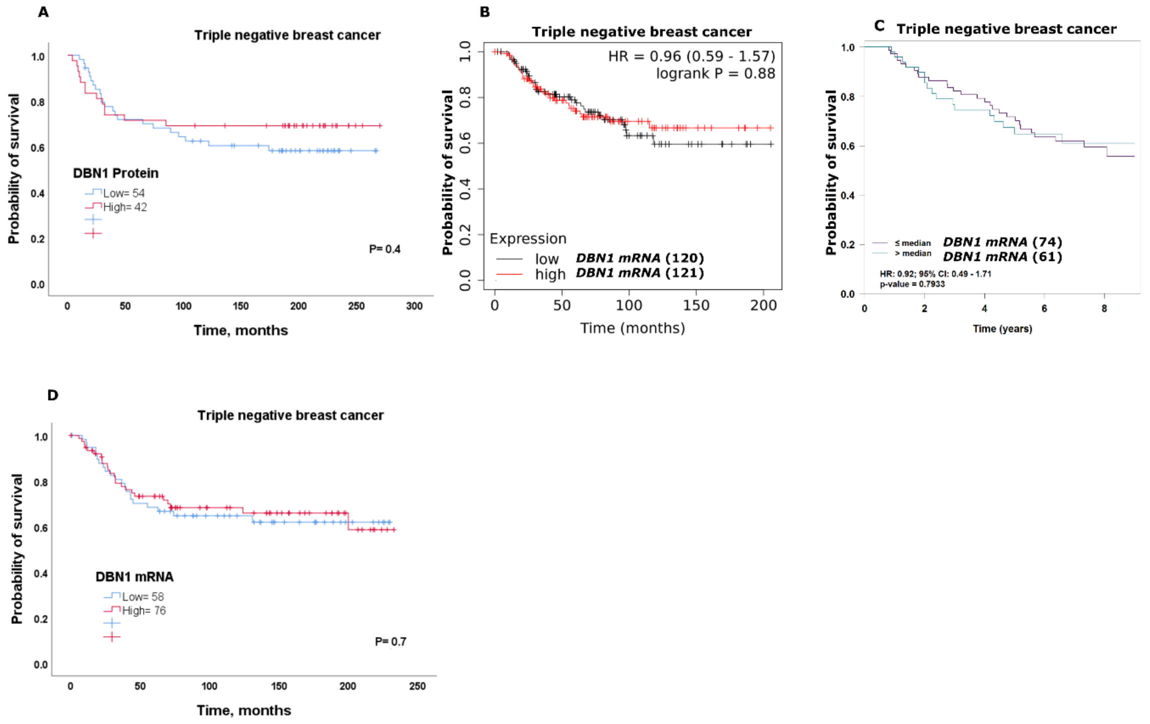

Figure S4. Kaplan–Meier of *DBN1 mRNA* expression and survival in triple negative breast cancer using the following datasets (**A**) METABRIC (**B**) KM-Plotter (**C**) bc-GenExMiner. (**D**) Kaplan–Meier of DBN1 protein expression and survival in triple negative breast cancer.

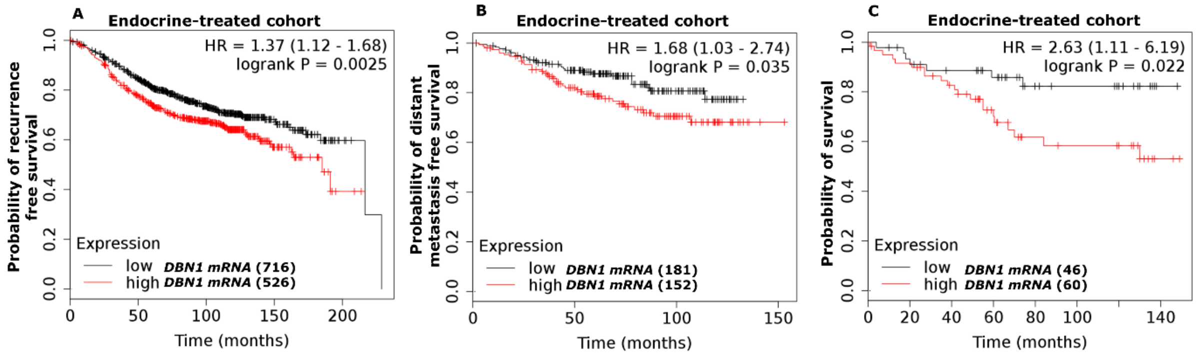

Figure S5. Kaplan-Meier of *DBN1 mRNA* expression in patients with luminal breast cancer who received adjuvant endocrine treatment using KM-Plotter dataset (**A**) Recurrence (**B**) Distant metastasis and (**C**) survival.

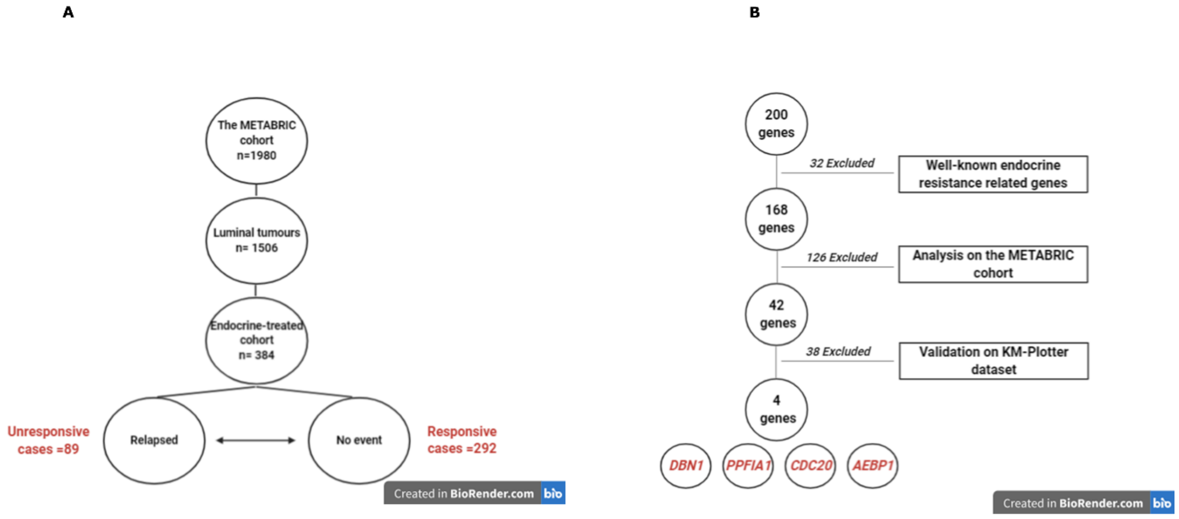

Figure S6. (**A**) Flow diagram illustrating the study design. (**B**) Flow diagram illustrating the filtering stages used to identify DEGs.

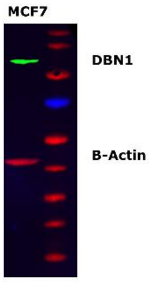

Figure S7. Western blotting result for DBN1 expression in MCF7 breast cancer cell lysate.

Table S1. Weighted Average Difference (WAD) statistics and ranking of the top DEGs associated with unresponsive cases to endocrine treatment.

Upregulated Genes			Downregulated Genes		
**Gene**	**WAD Statistics**	**WAD Ranking**	**Gene**	**WAD Statistics**	**WAD Ranking**
ACTA2	0.243137101	17	abParts	-0.314379611	3
ADAM15	0.120411754	151	ACTG1	-0.15722129	69
ADM	0.10959073	192	ADH1A	-0.11678724	163
AEBP1	0.264978283	11	ALDH3A2	-0.10994252	190
AGL	0.187732578	40	ANG	-0.118174842	161
APOC1	0.13237327	116	ANKRD30A	-0.133500516	112
APOE	0.149539483	79	BHLHE40	-0.205332849	31
ATOX1	0.121073855	149	BTG2	-0.163421473	65
BAMBI	0.194742652	35	C10orf116	-0.222339858	23
BGN	0.203583857	32	C17orf58	-0.114490894	174
C4orf48	0.117917975	162	CCL19	-0.144969152	91
C5orf46	0.121081909	148	CFD	-0.131432247	118
C7orf10	0.126572144	129	CLIC6	-0.155034397	73
CCNB2	0.133962205	110	CRTAP	-0.116134886	167
CCND1	0.176826971	47	CYBRD1	-0.109170617	194
CD24	0.25137787	14	DARC	-0.133563794	111
CDC20	0.208505122	29	DUSP1	-0.240647522	18
CDCA5	0.157028389	70	EEF1G	-0.120224837	153
CKS2	0.11622632	166	EGR1	-0.143199067	95
CNIH4	0.111428522	180	EGR2	-0.114499794	173

COL10A1	0.273231275	8	FOS	-0.290031548	7
COL11A1	0.311377881	5	FOSB	-0.226732301	21
COL1A1	0.267896706	9	GIMAP7	-0.119349281	156
COL1A2	0.173602661	49	GSTO2	-0.124334344	139
COL3A1	0.163990642	62	HBA2	-0.227061772	20
COL4A1	0.149121174	80	HBB	-0.209932824	28
COL5A1	0.169268472	55	HIST1H4C	-0.129914244	123
COL5A2	0.146108758	86	HLA-A	-0.130476597	122
COL8A1	0.171943296	50	HNRNPA1L2	-0.119329512	157
COMP	0.312142695	4	IER3	-0.150234817	78
CTTN	0.215048506	25	IGJ	-0.208179198	30
DBN1	0.115343993	169	IGLL1	-0.125570544	133
DENND1B	0.110403901	185	ITM2A	-0.185533092	41
DKK3	0.145850841	87	ITPR1	-0.119070668	158
DPYSL3	0.139158915	103	LRIG1	-0.111954551	177
EEF1A2	0.21693638	24	LYZ	-0.183522675	44
ENO1	0.154988314	74	MAOA	-0.155239476	72
ERBB2	0.140079066	101	MAPT	-0.163446629	64
EZR	0.183262994	45	MFAP4	-0.116721759	165
FAM38A	0.111546275	179	MGP	-0.125262772	135
FASN	0.11034215	187	NAT1	-0.125961415	131
FEN1	0.110005628	189	OSBPL1A	-0.129645778	124
FLJ40504	0.189555288	38	PRDX3	-0.137883023	106
FTL	0.145220353	90	PTGDS	-0.157893435	68
GAPDH	0.196842948	34	PTPLAD1	-0.151046154	77
GPI	0.139445228	102	RERG	-0.146664929	85
GRP	0.155826937	71	RPL22	-0.132441381	115
HIST1H1C	0.143257652	94	RPL27	-0.111225777	182
HLA-DRB1	0.124906721	138	RPS25	-0.107064464	198
HSPB1	0.166438633	59	RPS26P11	-0.131279982	119
IDH2	0.111159835	183	RPS28	-0.125042995	137
IFI27	0.222355759	22	SDCBP	-0.132662216	113
IGFBP3	0.106872137	200	SEPP1	-0.191455618	37
IGFBP5	0.126297072	130	SGK3	-0.106922143	199
ISLR	0.126814736	127	SLC39A6	-0.262802264	12
ITGA11	0.130566667	121	SLC7A2	-0.144753443	92
KIAA0101	0.126608334	128	SPARCL1	-0.127883643	126
KRT18	0.194556726	36	SRGN	-0.120200076	154
KRT7	0.261838689	13	SRP9	-0.134748906	109
KRT8	0.159321233	67	STC2	-0.166669702	58
LAD1	0.246385065	16	STOM	-0.120556989	150
LAMA5	0.121961933	143	SUSD3	-0.12549069	134
LASP1	0.111295375	181	TMBIM4	-0.118297895	160
LFNG	0.130717851	120	TMEM101	-0.109504953	193
LGALS1	0.12306019	142	TMEM99	-0.116748037	164
LUM	0.169870095	53	TSC22D1	-0.151416796	76
MARCKS	0.115931862	168	TSPAN3	-0.109025571	195
MCM7	0.114453669	175	UBB	-0.111742774	178
MFAP5	0.180437184	46	UGCG	-0.168365194	56
MFGE8	0.142270577	97	ZFP36	-0.138516844	105
MMP11	0.387014086	2	ZNF148	-0.115070433	170
MUC1	0.163815552	63			
MX1	0.107803387	197			
MXRA5	0.125805665	132			
MYC	0.132583238	114			
MYL6	0.145259516	89			
NCRNA00152	0.144037079	93			
NFKBIZ	0.162143882	66			

NOX4	0.110391774	186
NQO1	0.112987389	176
NUSAP1	0.12505333	136
PAFAH1B3	0.14532086	88
PAQR4	0.14058893	99
PHGDH	0.17076259	52
PITX1	0.138999423	104
PKM2	0.132130517	117
PLAU	0.175386193	48
PLS3	0.118635985	159
PMEPA1	0.12137618	146
POLD2	0.121381593	145
PPFIA1	0.148590232	82
PPP1R14B	0.140653682	98
PRC1	0.128867431	125
PRRX1	0.121868291	144
PSMB3	0.119964011	155
PTTG1	0.170776437	51
PTTG3	0.109619359	191
RARRES2	0.108432492	196
RPS6KB2	0.121087683	147
S100A16	0.166201275	60
S100A8	0.120286156	152
S100A9	0.143172754	96
S100P	0.571077669	1
SCGB2A2	0.265146337	10
SDC1	0.249860377	15
SEC61G	0.148553953	83
SERPINH1	0.184857184	42
SFRP2	0.123989769	140
SLC7A5	0.14906711	81
SPOCK1	0.135757552	107
SPP1	0.31014639	6
STIP1	0.123249991	141
SULF1	0.198185908	33
TACSTD2	0.183682573	43
TAGLN	0.114534593	172
TEAD2	0.16684977	57
TFF3	0.212899137	27
TGFBI	0.164341231	61
THBS1	0.134804144	108
THBS2	0.169287984	54
TMEM158	0.140549549	100
TMSB10	0.189481353	39
TOP2A	0.110248636	188
TPM2	0.213559405	26
TUBA1B	0.115054112	171
TYMP	0.110901695	184
UBE2C	0.233314012	19
UBE2S	0.148485119	84
X64709	0.151709333	75

Table S2. List of pathways associated with the DEGs associated with unresponsive cases to endocrine therapy.

ID	Pathway Name	Genes	*p* value
**P00034**	Integrin signalling pathway	12	0.01
**P00047**	PDGF signalling pathway	5	0.03
**P06959**	CCKR signalling pathway	8	0.1
**P00057**	Wnt signalling pathway	5	0.5

Table S3. Association of DBN1 expression and clinicopathological parameters in luminal breast cancer.

	***mRNA* expression**				**Protein expression**			
	**Low**<br>No. (%)	**High**<br>No. (%)	*p*	*p**	**Low**<br>No. (%)	**High**<br>No. (%)	*p*	*p**
**Tumour Size (cm)**								
**< 2 cm**	71 (68.9)	32 (31.1)	0.2	0.2	129 (53.5)	112 (46.5)	0.6	0.8
**≥ 2 cm**	68 (60.7)	44 (39.3)			106 (55.5)	85 (44.5)		
**Tumour Grade**								
**1**	68 (84.0)	13 (16.0)	0.001	0.003	41 (59.4)	28 (40.6)	0.4	0.9
**2**	196 (73.7)	70 (26.3)			105 (55.9)	83 (44.1)		
**3**	163 (64.2)	91 (35.8)			91 (51.1)	87 (48.9)		
**NPI**								
**GPG**	170 (82.5)	36 (17.5)	0.00006	0.0004	92 (59.4)	63 (40.6)	0.2	1.4
**MPG**	216 (66.7)	108 (33.3)			109 (51.2)	104 (48.8)		
**PPG**	52 (62.7)	31 (37.3)			34 (53.1)	30 (46.9)		
**Nodal Stage**								
**1**	90 (68.7)	41 (31.3)	0.2	0.3	150 (56.4)	116 (43.6)	0.5	0.8
**2**	41 (59.4)	28 (40.6)			69 (52.3)	63 (47.7)		
**3**	8 (53.3)	7 (46.7)			18 (48.6)	19 (51.4)		
**Vascular Invasion**								
**Negative**	91 (68.4)	42 (31.6)	0.1	0.2	147 (54.4)	123 (45.6)	0.9	1.0
**Positive**	49 (59)	34 (41.0)			87 (54.0)	74 (46.0)		
**Progesterone Receptor**								
**Negative**	120 (70.2)	51 (29.8)	0.4	0.4	54 (59.3)	37 (40.7)	0.3	1.0
**Positive**	313 (73.0)	116 (27.0)			182 (53.5)	158 (46.5)		

*p**: Adjusted *p* values, NPI: Nottingham Prognostic Index; GPG: Good prognostic group; MPG: Moderate prognostic group; PPG: Poor prognostic group.

Table S4. Clinicopathological characteristics of luminal breast cancer cohorts.

**Parameters**	**METABRIC Cohort**<br>***mRNA***	**Nottingham Cohort**<br>**Protein**
	**No. (%)**	**No. (%)**
**Age**		
< 50	228 (15)	449 (31)
≥ 50	1278 (85)	1006 (69)
**Tumor size (cm)**		
< 2 cm	475 (31.5)	806 (55.7)
≥ 2 cm	1031 (68.5)	640 (44.3)
**Tumour grade**		
1	166 (11.5)	388 (24.7)
2	707 (49.1)	661 (42.1)
3	565 (38.4)	522 (33.2)
**Nottingham Prognostic Index**		
GPG	623 (41.3)	598 (41.4)

MPG	772 (51.2)	668 (46.1)
PPG	111 (7.5)	180 (12.5)
**Nodal stage**		
1	404 (36.2)	1025 (65.1)
2	634 (56.8)	439 (27.9)
3	78 (7)	111 (7)
**Vascular invasion**		
Negative	N/A	1000 (69)
Positive		439 (31)
**Endocrine therapy alone**		
No	234 (15.5)	810 (50)
Yes	384 (25.5)	554 (35)
Other*	888 (59)	237 (15)
**Recurrence**		
No	496 (33)	953 (60)
Yes	118 (7)	618 (38)
Unknown	892 (60)	30 (2)
**Distant metastasis**		
No	476 (31)	1079 (67)
Yes	143 (10)	514 (32)
Unknown	887 (59)	8 (1)
**Progesterone receptor**		
Negative	486 (23.2)	300 (21.3)
Positive	1020 (76.8)	1103 (78.7)

GPG: Good prognostic group; MPG: Moderate prognostic group; PPG: Poor prognostic group, * Include patients who received chemotherapy alone or combination of chemotherapy and endocrine therapy.

© 2020 by the authors. Licensee MDPI, Basel, Switzerland. This article is an open access article distributed under the terms and conditions of the Creative Commons Attribution (CC BY) license (http://creativecommons.org/licenses/by/4.0/).
